# Benefits and Risks of Preventing Twin Pregnancies

**DOI:** 10.3390/ani11010148

**Published:** 2021-01-11

**Authors:** Irina Garcia-Ispierto, Fernando López-Gatius

**Affiliations:** 1 Agrotecnio Centre, 25198 Lleida, Spain; irina.garcia@udl.cat; 2 Department of Animal Science, University of Lleida, 25198 Lleida, Spain; 3 Transfer in Bovine Reproduction SLu, 22300 Barbastro, Spain

**Keywords:** twinning, additional corpus luteum, pregnancy loss, GnRH, hCG, bovine

## Abstract

**Simple Summary:**

While cows usually give birth to singletons, the incidence of twin births has increased considerably during the past few decades alongside milk production. In most cases, multiple pregnancies arise from the simultaneous formation of two or more ovulatory follicles in either the same ovary or both ovaries. Twin pregnancies have devastating effects on cow welfare and the economy of dairy herds. To prevent them, strategies proposed have been the transfer of a single female beef cow embryo produced in vitro to a cow that is not suitable for producing replacements, or the drainage of co-dominant follicles at insemination to prevent twin pregnancies in cows with genetic merit. Developing strategies to reduce the incidence of multiple ovulations and twin pregnancies should be a main focus of clinicians responsible for reproduction in dairy herds. The two procedures mentioned could be components of a weekly reproductive control program and applied to synchronized cows. As a result, embryo survival should improve, avoiding economic losses associated with twin pregnancies, and beef output from the herd will accordingly increase. Last but not least, if twin pregnancies are prevented, the cow’s general health and welfare state will certainly improve.

**Abstract:**

Clinical problems associated with twin pregnancies have been well established, and twin births are now considered undesirable or even disastrous for the dairy cattle industry and the individual cow. The high incidence of early fetal loss, abortion during the mid-lactation period, dystocia, stillbirth, and placenta retention should be considered a preventable consequence of management, as these disorders greatly compromise the welfare and productive lifespan of a cow carrying or delivering twins. The use of sexed semen generates herd replacements and additional heifers, so a proposed strategy for twin pregnancy prevention is the transfer of a single in vitro-produced female beef cow embryo to cows not suitable for producing replacements. Another proposed strategy is drainage at insemination of co-dominant follicles to prevent twin pregnancies in cows with genetic merit. As a result, embryo survival should improve, economic losses associated with twin pregnancies will be prevented, beef output from the herd will be increased, and the health and welfare of the cow will certainly benefit. In this review, the clinical prospects of preventing or avoiding twin pregnancies using both procedures are discussed.

## 1. Introduction

Twin pregnancies and subsequent twinning are considered highly undesirable in the dairy cattle industry and also from the perspective of the cow. The high incidence of early fetal loss [1,2], abortion during the middle period of lactation [3,4], dystocia, stillbirth, and placenta retention [5,6,7,8] could be considered a preventable consequence of management, as these disorders greatly compromise the welfare and productive lifespan of a cow carrying or delivering twins [8,9]. In some herds, the twinning rate may exceed 12% [10], and 10% of cows deliver twins at least once during their life [8]. An example of the economic impact of twin pregnancies is an annual cost estimate in the United States of 96 million USD [11]. Although hormone treatment or induced twin reduction at pregnancy diagnosis may mitigate the negative effects of twin pregnancies [2,12], the adage “prevent is better than cure” [13] is entirely appropriate for this problem.

Over the past three decades, multiple ovulation rates and thus the incidence of twin pregnancies and twinning have increased together with milk production [2]. The incidence of multiple ovulations increases with age and with genetic, nutrition, and management improvements related to increased milk production [2,8]. In addition, this is because of synchronization protocols for fixed-time artificial insemination (FTAI), which have become an essential component of the management of dairy cow reproduction. Some of these FTAI protocols increase the twin pregnancy rate [14]. Therefore, a goal of such protocols should be to avoid or prevent multiple ovulations. For example, it has been shown that by shortening the time of treatment [15,16] and increasing progesterone before FTAI [17,18], twin pregnancies can be significantly reduced. However, although these results are encouraging for the development of protocols to reduce twins, the causal mechanisms of multiple ovulations are not well understood [19]. Recently, two strategies, the transfer of a single embryo produced in vitro and follicular drainage of co-dominant follicles at insemination, have been proposed to prevent twin pregnancies [20,21]. In this review, the clinical prospects and weaknesses of both procedures along with possible improvements are discussed.

## 2. Transferring a Single In Vitro Produced Embryo

The global use of in vitro-produced embryos (IVP) has increased over the past decades, surpassing the number of in vivo-produced embryos since 2016 [20,22]. While the main interest of IVP embryos lies in genetic gain, their lower costs and an increased efficiency of procedures means they are the most effective method to improve fertility during periods of heat stress [23,24,25] and for repeat-breeder cows following AI [26,27,28,29]. In effect, a single developing blastocyst transferred into the uterine horn ipsilateral to the corpus luteum (CL) does away with the risk of the in vivo fertilization of two or more oocytes following insemination, particularly in older cows. Twin pregnancies are more frequent in multiparous cows and may account for 25% of all pregnancies during the early fetal period in cows in their third lactation or more [3]. Many technicians are skilled at embryo transfer procedures and could in some circumstances replace AI with the transfer of an IVP embryo.

### 2.1. Benefits

As the use of sexed semen in heifers offers the benefit of herd replacements and additional heifers [29], embryo transfer of a single beef cow embryo to cows not appropriate for replacements should increase herd profitability. Sexed semen is also used in IVP procedures [30,31] so that by transferring a female beef cow embryo, the incidence of male calf-related dystocia will be reduced, and milk production increased. Indeed, the birth of a female calf has been associated with a milk production increase [32,33]. A further benefit of transferring beef cow IVP embryos should be increased beef output from dairy herds, making land use more efficient than when rearing beef cow herds with the consequence of reducing greenhouse gas emissions [34]. However, the efficiency of the in vitro production of embryos leaves much scope for improvement. Only 27% of cattle receiving IVP embryos produce a live calf [35] and these calves are more susceptible to experiencing large offspring syndrome compared with in vivo-generated newborns [36,37,38,39]. Fetal overgrowth syndrome induced by assisted reproduction has also been described in humans [39]. Further, a series of abnormalities such as reduced preimplantation energy metabolism [40] and chromosome aberrations [41,42] and embryo development defects, or young fetuses and placentas [43,44,45] can explain both individually and collectively the extremely high risk of such pregnancies [46,47]. Failure of a pregnancy with an IVP embryo ranges from 59% to 85% and seems to increase using sexed semen [35]. While the literature regarding the use of sexed semen in IVP is limited [22], a high pregnancy loss rate would be expected using sexed IVP embryos, as this has been described in heifers after AI with sexed semen [48]. In a recent study on 1562 heifers receiving a fresh embryo in vitro produced using conventional semen, GnRH treatment on Day five post-estrus (one to three days before embryo transfer) increased the formation of additional corpora lutea and reduced the pregnancy loss rate recorded on day 60 of gestation [49].

### 2.2. Risks

Regardless of the production of high genetic index calves, a main target of the IVP embryo industry is the use of IVP embryos under heat stress conditions [23,24,25] or in repeat-breeder cows [26,27,28,29]. In this context, preventing twin pregnancies through the transfer of a single embryo could be an important therapeutic approach. However, specific studies have yet to be carried out. After the transfer of an in-vivo-produced bovine embryo, the occurrence of monozygotic twins has been described [50,51], with an incidence of 1.6% after the transfer of a single in vitro-produced equine embryo [52], and extensive reports existing in humans. Assisted reproductive technologies have increased the incidence of multiple pregnancies in women, with rates of 1.4% to 13.2% reported for monozygotic twins [53,54,55], and of 0.04% to 0.3% for triplets [56,57]. Even a monozygotic quadruplet has been observed [57]. The first issues that need to be investigated are the incidence of twins after the transfer of a single IVP embryo and possible risk factors related to monozygotic twins.

## 3. Puncture and Drainage of the Smaller Co-Dominant Follicles

**Figure 1:**
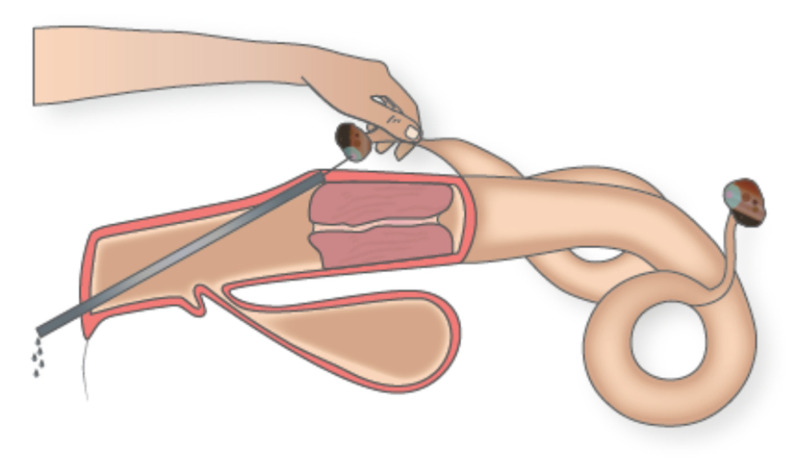
Follicular puncture and drainage with no aspiration of the smaller co-dominant (ovulatory) follicle using a transvaginal hand-guided metallic cannula in a cow with a follicle of pre-ovulatory size (blue) in each ovary. Drawing by López-Gatius. The color artwork is courtesy of Cris Segú Mora.

Most twin pregnancies derive from multiple ovulations which result from the simultaneous formation of two or more co-dominant follicles either in one or both ovaries [58,59]. The incidence of multiple ovulations in high producers at insemination may exceed 20% [60,61,62,63] and the rate of having two or more co-dominant follicles at the time of AI may be over 50% in cows subjected to a FTAI protocol [64]. Therefore, emptying of all follicles of pre-ovulatory size except the largest should prevent twin pregnancies. In effect, there is already evidence that transvaginal puncture and drainage of the smaller follicle at the time of insemination in cows with two co-dominant (ovulatory) follicles eliminates the risk of twin pregnancy without reducing fertility [65,66,67,68]. Drainage of follicles may be either ultrasound-guided [65,66], or hand-guided using a steel cannula designed for follicular cyst puncture [67,68]. This instrument makes the procedure quick and easy for an experienced technician after the detection of follicles of pre-ovulatory size by rectal palpation (Figure 1).

### 3.1. Benefits

**Figure 2:**
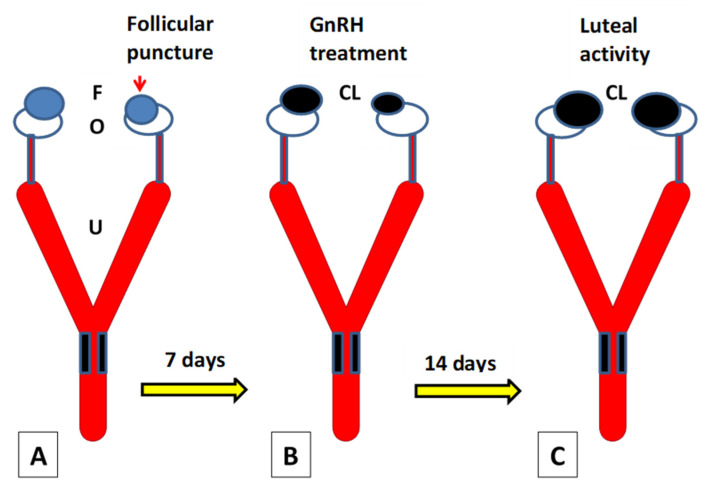
Puncture and drainage of the smaller co-dominant follicle at the time of insemination in cows with a follicle of pre-ovulatory size in each ovary (**A**). GnRH treatment is given seven days post-drainage to reinforce luteal activity of the drainage-induced luteal tissue (**B**). Luteal activity determinations are made 21 days post-drainage (**C**). Both corpora lutea are not distinguishable at this time point [65]. F: follicles; CL: corpora lutea; O: ovaries; U: uterus.

Ablation or removal of the dominant follicle by ultrasound-guided transvaginal follicle aspiration has been extensively used to enhance the super-ovulatory response in embryo transfer programs in cattle and buffalo [69,70,71,72]. The follicular drainage procedure referred to here avoids suction of the antral fluid. Drainage with no aspiration leaves a sufficiently large number of granulosa cells in the follicle for the subsequent formation of luteal tissue [66]. All drained follicles develop as a CL seven days post-drainage [65,66,67,68]. GnRH treatment at this point, seven days post-estrus, reinforces the function of these induced luteal structures (Figure 2) so that drainage-induced CL and fellow CL are similar in terms of size and vascularization as determined through Doppler ultrasonography [66]. The drainage-induced additional CL has been shown to favor embryo survival [67] in a similar way to an additional CL following spontaneous ovulation, and this has proved to be a very strong factor reducing the risk of pregnancy loss during the late embryonic/early fetal period [73].

### 3.2. Risks

It should be noted that the follicular drainage procedure has an important shortcoming that needs improving. Although fertility is similar in drained and non-drained cows, a high percentage of non-drained follicles fail to ovulate. Based on data compiled from our four latest studies [65,66,67,68], in 23.3% (70/300) of follicle-drained cows, the non-drained follicle never reached the stage of ovulation. Pregnancy was not detected in these cows, whereas the pregnancy rate was similar for all drained (29.7%: 89/300) and non-drained (31.5: 95/302) cows. Hence, the compromised fertility of the whole sample of drained cows was offset by a high pregnancy rate of ovulating drained cows (38.7%: 89/230). The incidence of ovulation failure of the non-drained follicles and the subsequent high pregnancy rate of the ovulating ones need to be clarified. Treatment at drainage with a strong inducer of ovulation such as human chorionic gonadotropin (hCG) could probably improve the ovulation rate of the non-drained follicles. However, caution is needed with this type of treatment. Follicles smaller than 10 mm, that is, of pre-ovulatory size [74], are able to respond to hCG treatment [75] and this may reverse the capacity of follicular drainage to prevent multiple pregnancies.

## 4. Conclusions

Follicular puncture and drainage with no aspiration and single embryo transfer may eliminate the risk of a twin pregnancy with the consequence of improved cow health and welfare. Following GnRH treatment, five to seven days post-estrus, both procedures promote the formation of an additional corpus luteum, and so reducing the risk of subsequent pregnancy loss. As older cows are the main population at risk of multiple ovulations, cows in their third lactation or more with no genetic merit could receive a single beef cow female embryo, whereas cows with a high genetic index could be inseminated with sexed semen following follicular puncture of co-dominant follicles. This strategy should increase herd profitability. Major concerns that still need to be addressed are the possible risks of the transfer of embryos produced in vitro using sexed semen and the low ovulation rate of non-drained follicles following follicular drainage.
